# COVID-19 and Protracted Displacement: a Scoping Review of Migration Policies in Mexico and Central America

**DOI:** 10.1007/s12134-023-01040-w

**Published:** 2023-05-11

**Authors:** Noor J. ten Have, Kassandra J. Jimenez, Jonas Attilus, Maria B. Livaudais, Brittney S. Mengistu

**Affiliations:** 1 Athena Institute, Vrije University Amsterdam, 1081 HV Amsterdam, Netherlands; 2 Department of Psychiatry & Behavioral Sciences, University of Minnesota Medical School, Minneapolis, MN USA; 3 Department of Public Health, California State University East Bay, Hayward, CA USA

**Keywords:** COVID-19, Refugees, Policies, Central America, Mexico, US, Noor J. ten Have and Kassandra J. Jimenez are co-first authors

## Abstract

By the end of 2020, more than 500,000 migrants from Central America, Haiti, Africa, and Asia sought asylum along the US-Mexico border despite COVID-19-related travel restrictions and public health measures. A scoping review was conducted to understand the role of COVID-19-related policies on irregular migration flows through Central America and Mexico and to examine the experiences of asylum seekers traversing this region. Peer-reviewed literature, policy briefs, and commentaries were screened for inclusion, resulting in 33 documents selected for this review. This review identified three dominant themes: border closures due to multiple national migration policies, delays in asylum procedures, and increased risks to migrant wellbeing. This article argues that border closures were a punitive policy measure to deter irregular migration during the COVID-19 pandemic. Implications for future research and policy include prioritizing the health needs of asylum seekers and advocating the appropriateness and effectiveness of immigration and public health policy.

## Introduction

In September 2021, images of US border patrol agents on horseback forcefully preventing Haitian migrants from crossing the US-Mexico border circulated national and international media outlets (Kosman, 2021). These images not only sparked international outrage regarding the violent practices used to deter irregular migration but also highlighted the current policies, albeit less known, that justify violent measures in the policing of irregular migration. Despite these violent policing practices and restrictive immigration policies, irregular migration to the US-Mexico border remains at an unprecedented high (MDP, 2021). By the end of 2020, more than 2.8 million refugees and asylum-seekers were in the Americas (UNHCR, 2021a), with approximately 432,700 migrants from El Salvador, Guatemala, and Honduras seeking asylum in the US and 78,600 migrants from these three countries seeking asylum in Mexico despite COVID-19-related travel restrictions and public health measures (UNHCR, 2021b). According to UNHCR (2021b), these statistics represent a substantial decrease in asylum claims due to public health-related movement restrictions. The US documented a 75% decrease in family arrivals and 4% decrease in unaccompanied children, and Mexico also noted a 41% decrease in overall asylum claims but a 44% increase in unaccompanied children (ibid).

Central America, Mexico, and the US continue to receive an influx of irregular migrants fleeing violence, humanitarian disasters, and economic instability (MDP, 2021). Irregular migration is the “movement that takes place outside the regulatory norms of the sending, transit and receiving country” (IOM, 2019). Irregularity can take several forms like residing in a country with a denied asylum application, overstaying visa periods illegally, irregular employment agreements, crossing borders undocumented, and so on (Vespe et al., 2017). This form of migration not only encompasses passing borders without legal documentation but also relates to the fragile and fluctuating status of irregularity that migrants are subjected to in various transit countries due to the abrupt and ever-changing policies and laws (IOM, 2019; MDP, 2021).

Examining these migration patterns in more detail, this article investigates the impact of COVID-19-related policies and restrictions on irregular migration in Central America and Mexico. The following section details the background of the scoping review, specifically the context of irregular migration within Central America and Mexico and the policies that governed mobility prior to COVID-19. The next section explains the methodological approach to conducting this scoping review and is followed by the results. The findings provide in-depth insight into existing peer-reviewed literature and policy briefs on COVID-19, and migration policies implemented at the onset of the pandemic. The discussion argues that the themes presented in the results are increasingly relevant to current public health and migrant policy debates, as emerging and unprecedented migration flows impact all corners of the world. The paper concludes with drawing attention to migrant experiences traversing this region and the gradual, yet stark contrast in the increased implementation of restrictive migration policies that do not sufficiently account for their wellbeing.

## Background

### Interregional Migration Flows

Guatemala, El Salvador, and Honduras, commonly referred to as the Northern Triangle, experienced a dramatic increase in violence, crime, and narco-trafficking in recent years, leading to the forced migration of almost 2 million individuals to North America (Orozco, 2018). Over the past two decades, the homicide rates in these countries have become among the highest in the world (Cheatham et al., 2019). For example, in 2018 El Salvador’s homicide rate was 52 per 100,000 people, and Honduras had a rate of 38 per 100,000 people (World Bank, 2022). This violence disproportionately affects women, as Honduras and El Salvador had the highest femicide rates in Latin America in 2019 (CEPAL, 2020). In addition to fleeing violence, individuals are also fleeing environmental and humanitarian crises like back-to-back hurricanes in Central America and the Caribbean, impacting more than 7.5 million people in 2020 (Pulwarty et al., 2022). Further, the sociopolitical instability of countries like Haiti has exacerbated existing environmental vulnerabilities, like regularly occurring earthquakes and natural disasters (Felima, 2009). These environmental conditions have resulted in an unprecedented movement of Haitian migrants to Latin America (Audebert, 2017). For example, in 2018, approximately 7,000 Haitian migrants were residing in towns along the US-Mexico border, constituting the largest migrant group aside from Central American and Mexican migrants crossing into the US (Adossi et al., 2018).

The agriculture sector in Central America has also been affected by climate change, like destructive coffee rust, a fungus in coffee plants that negatively impacts vulnerable economies and precipitates food insecurity throughout Central America (Cheatham et al., 2019). Interregional migration has also occurred due to poor economic and political conditions. Since April 2018, the political turmoil in Nicaragua has forced more than 100,000 Nicaraguans to the neighboring countries of Costa Rica and Panama in search of safety and employment (IOM, 2021; Orozco, 2018). Though migration within Central America remains high, thousands of migrants continue to irregularly migrate north to reach the US. These migrants have either accumulated on Mexico’s southern border with Guatemala or the northern border with the US (IOM, 2019). The number of migrants from Central America seeking international protection in Mexico in 2019 was over 500,000 during the first half of the year, which was almost 20 times higher than the number of irregular migrants in 2015 (UNHCR, 2021a).

### Extracontinental Migration Flows

Irregular migration throughout Central America has recently seen a growth in extra-continental migration with individuals from Asia and sub-Saharan Africa (IOM, 2021). With the tightening of migration policies in the European Union (E.U.), many of these migrants have turned to the Americas for refuge. Before August 2019, several South American countries had minimal visa restrictions, such as Ecuador, Brazil, and Guyana, which allowed Asian and African migrants to lawfully enter the country and stay for 90 days (Priya Morley et al., 2021; Yates, 2019). The route to the US-Mexico border typically originates in Brazil, continues to Peru, then Ecuador, Colombia, Panama, and Costa Rica, and continues north to the US. According to Panama’s Servico Nacional de Migracion (National Migration Agency), the number of African and Asian migrants crossing through the Darien Gap has increased by 715% from 2014 to 2019 (MDP, 2021). Similar to Central Americans traversing this route, extracontinental migrants have reportedly recruited human smugglers to facilitate their journey, often paying between $2,500 and 13,000 USD to transit from Brazil to Mexico (Priya Morley et al., 2021).

Between 2010 and 2019, more than 31,500 African and 30,400 Asian irregular migrants were apprehended by Mexican authorities (Campos-Delgado, 2021). According to Priya Morley et al. (2021), the majority of African migrants detained by Mexico’s Instituto Nacional de Migración, (National Institute for Migration or INM) authorities are from Eritrea, Ghana, Somalia, Cameroon, and the Democratic Republic of Congo (DRC). The political instability, violence, persecution of LGBTQ communities, and economic deprivation in these African countries push migrants to search for refuge through new migration routes, which entails traversing through the Americas to the US or Canada (Yates & Bolter, 2021). The majority of Asian migrants are reportedly from India, Bangladesh, and Nepal, with Indian migrants constituting almost 9,000 apprehensions at the US-Mexico border in 2018 (Yates, 2019). Unlike their African counterparts, Asian migrants flee for economic reasons or natural disasters.

Campos-Delgado’s (2021) analysis of extracontinental migration flows in Mexico reveals that Africans and Asians constitute a fraction of irregular migrants, but unlike Central Americans, extracontinental migrants are subject to unsystematic migration arrangements in Mexico. Though fewer African and Asian migrants were deported from Mexico compared to Central Americans, systematic barriers such as long asylum proceedings have resulted in migrants abandoning their application to continue north to the US. According to Campos-Delgado, more extracontinental migrants are issued ‘self-deportation’ arrangements, allowing migrants to freely traverse the country with the ultimate directive to exit the country. Campos-Delgado argues that the high abandonment rate (74% Asians; 58% Africans) may be attributed to the desire to seek asylum in the US, poor migration governance in Mexico, or other socioeconomic constraints. Additional reasons for abandonment may include precarity and threats to individual health, such as difficulties accessing clean water, and limited access to healthcare services (Hernandez-Arriaga & Domínguez, 2020).

### US and Mexican Migration Policies Pre-COVID-19

Before 2018 and the COVID-19 pandemic, Mexico’s policies on immigration and refugees were regarded for their solidarity and generosity, although not without criticism (Vega, 2021). Mexico signed several protocols and conventions on refugees and migrations such as the 1951 Geneva Convention, 1967 New York Protocol and 1984 Cartagena Declaration on Refugees (protection of Latin America migrations). Many of the country’s immigration laws also grant protection for people whose freedom and/or security are threatened as well as provide a framework for migrant rights (Vega, 2021). The 2018 ‘Central American migrant crisis’, also known as the year of the caravans, consisted of thousands of Central American migrants travelling in caravans from Guatemala, El Salvador, and Honduras (the Northern Triangle) to the US (UNHCR, 2022), and marked the gradual implementation of restrictive migration policies in Mexico (Arriola Vega, 2021).

In 2018, the US enacted “Metering,” which limits the number of asylum claims lodged per day and ultimately prolongs the wait of asylum seekers hoping to cross the US-Mexico border (Leutert, 2020a, 2020b). That same year the Trump Administration threatened tariffs on Mexican goods to pressure the Mexican government to reform its migration policies. In response, Mexico adopted the Migrant Protection Protocols (MPP, Remain in Mexico Policy) in early 2019, which requires all returnees to exit through the southern border (Soto, 2020). Additionally, Soto’s (2020) policy brief describes how the US and Mexico signed a joint declaration in 2019 to further minimize irregular migration flows between the two countries. This declaration contains five key obligations (pg. 4):

1. Mexico will strengthen its migration control at the Mexico-Guatemala border by deploying the Mexican National Guard.
2. Mexico will accept more non-Mexican asylum seekers deported by the US and commit to increasing humanitarian protections.
3. The US will expedite the processing asylum cases
4. Mexico and the US will cooperate to tackle and dismantle human-smuggling networks.
5. Mexico and the US will commit to addressing migration through development and economic investment in southern Mexico and Central America.

During the initial implementation of MPP, Mexico did not claim responsibility for the program and framed it as a unilateral decision by the US. The Mexican government framed its role as being solely on the well-being of migrants and omitted mentioning their meeting with the US (Leutert, 2020a, 2020b). Further, Leutert (2020a, 2020b) asserts that Mexico allegedly refused to accept returnees at the beginning of MPP, but they have since adopted the policy to uphold their obligations. Per the declaration of the economic development in southern Mexico and Central America, the US is contributing $5.8 billion to support institutional reforms and economic development of the Northern Triangle (US Embassy in El Salvador, 2018). In December 2021, the US and Mexico launched the “Sowing Opportunities” Program to address the root causes of irregular migrants from the Northern Triangle (The White House, 2022). The adoption of this policy and the militarization of Mexico’s southern border with Guatemala illustrates a significant change in Mexico’s previously welcoming attitude and policies regarding asylum seekers. The increase in the militarization of borders has also been portrayed as means of deterring human smuggling. For example, Meyer and Isacson (2019) argue that Mexico is creating an intangible wall, or the “Wall before the Wall,” as a result of the US materializing the physical barrier along the US-Mexico border. It can be argued this shift in migration policy highlights how Mexico is not only willing to cooperate with the US to limit migration flows to the border but to also implement similar policies that limit migration into southern Mexico. Scholars have argued that the former US administration used Mexico as the “de facto wall” to minimize the influx of migrants from Central America (Riggirozzi, 2020). Moreover, the Asylum Cooperation Agreement between the US and Guatemala authorizes the US to deport asylum seekers to Guatemala, including nationals from the other Northern Triangle countries (Garrett, 2020). These trends in anti-migration policies illustrate how the US has been able to control the flow of migration in North and Central America leveraging their global position and resources while showing little regard for the countries in the region, their people, and irregular migrants.

Irregular migrants traversing Central America are extremely vulnerable to experiencing discrimination, violence, extortion, human smuggling, and other crimes because of their precarious migration status and living conditions (Faret et al., 2021). Many of these migrants live in constant fear of deportation and have limited access to health care services and legal employment possibilities (Faret et al., 2021). The COVID-19 pandemic and the associated policies have exacerbated the vulnerabilities of irregular immigrants in Central America (Bojórquez et al. 2021b), yet literature reviews on the effects of political decisions regarding COVID-19 on irregular migration in Central America and Mexico remain scant. Likewise, the experiences of migrants stranded in Central America and Mexico due to migration and health policies have not been systematically assessed to inform policymakers and researchers examining the fundamental needs of irregular migrants. Building on these notions, this literature review examines the impact of COVID-19-related policies and restrictions on irregular migration in Central America and Mexico*.* Two research questions guided this review: 1) How have COVID-19-related policies and restrictions in Central America, Mexico and the US impacted irregular migration flows to the US-Mexico border; 2) What are the experiences of irregular migrants stranded in Central America and Mexico as a result of these interregional COVID-19 policies and restrictions?

The following sections provide an overview of the methods used in this scoping review, followed by the results thematically identified as border closures, delays in asylum procedures and the wellbeing of migrants in detention centers. The discussion then situates the review findings into the broader discourses on migration and public health policies and its implications on migrant health, and concludes with a call to action for policymakers and practitioners working at the intersection of migration policy and health.

## Methods

This section describes the methodological approach taken in this scoping review and outlines the study design, the search strategy and selection of the sources, the data extraction and quality appraisal, data synthesis and the final eligibility criteria of this scoping review.

### Study Design

Due to the dearth of literature on this topic, a scoping review was considered the most appropriate approach to synthesizing the literature on the impact of COVID-19-related policies on irregular migration flows in Central America and Mexico. The structure and content of the methodology of this scoping review were guided by the Joanna Briggs Institute (JBI) Manual for Evidence synthesis 2021 and checked for appropriateness with the PRISMA for Scoping Reviews Checklist.

### Search Strategy and Selection of Sources

A systematic search strategy was used in Scopus (*n* = 157), Web of Science (*n* = 209), and ScienceDirect (*n* = 77). All digital sources were imported and assessed for inclusion with the use of Rayyan by two study authors. Articles were included if they included information on irregular migration during the COVID-19 pandemic and within the geographical restrictions of Central America, Mexico, and the US-Mexican border (Table 1). Studies were included if they were published between November 2019 and November 2021. Only sources written in English and Spanish were included due to the authors’ English and Spanish fluency. Exclusion criteria included studies published pre-COVID-19, regular migration, and geographical areas outside of the scope of this review. The Migrant Policy Institute publications database was searched to identify grey literature, such as policy briefs and governmental reports. Moreover, relevant organizational websites were searched like the International Organization for Migration and Migration data Portal. Other literature sources were identified through reference screening of the included articles. The first five sources were screened together to validate the inclusion and exclusion criteria, thereafter, the screening process occurred separately with an equal division between the number of identified sources. In case of disagreement during this screening process, the two reviewers discussed the discrepancies in the assessment of the sources until an agreement was reached.

**Table 1:** Eligibility criteria

Inclusion/exclusion criteria	
Population	Included: irregular migrants, refugees, asylum seekers, undocumented, forced migrants Excluded: regular migrants or workers
Concept	Included: COVID-19, pandemic Excluded: before 2019, unrelated to COVID-19
Context	Included: Central American countries, Mexico, US-Mexican border Excluded: All other locations
Study design	Included: All, if meeting inclusion criteria

### Data Extraction and Quality Appraisal

A data extraction sheet was created to collect the citation details, population, context, geographical location, study design, research question, migrant experiences, policies and migration flows of each article. Sources were simultaneously assessed on its quality and reliability for inclusion. All articles were assessed using the Critical Appraisal Skills Programme qualitative appraisal tool, which examines the articles’ quality and appropriateness for inclusion in the review. Grey literature was assessed by the A.C.C.O.D.S. tool, which focuses on to what extent these sources are reliable, valid and appropriate for this review. None of the articles nor grey literature documents were disregarded based on the quality appraisal process.

### Data Synthesis

Thematic analysis was performed jointly by two study authors in a basic coding process after data extraction. First, codes were identified through iterative inductive and deductive coding. Inductive codes, for example, included the US-Mexican border, violence, asylum-seeking, family separation, border control/closure, and detention centers. Deductive codes were linked to the review research concepts, like COVID-19 migration policies, Central American irregular migration flows, and migrant experiences. Additionally, all migration policies were grouped by three overarching policy domains; border closures, asylum procedures, and livelihoods. Migrant experiences and migration flows were linked to one of the three domains, allowing authors to identify and assess the relationship between codes and emergent themes (Braun & Clarke, 2006).

### Final Eligibility Criteria

**Fig. 1:**
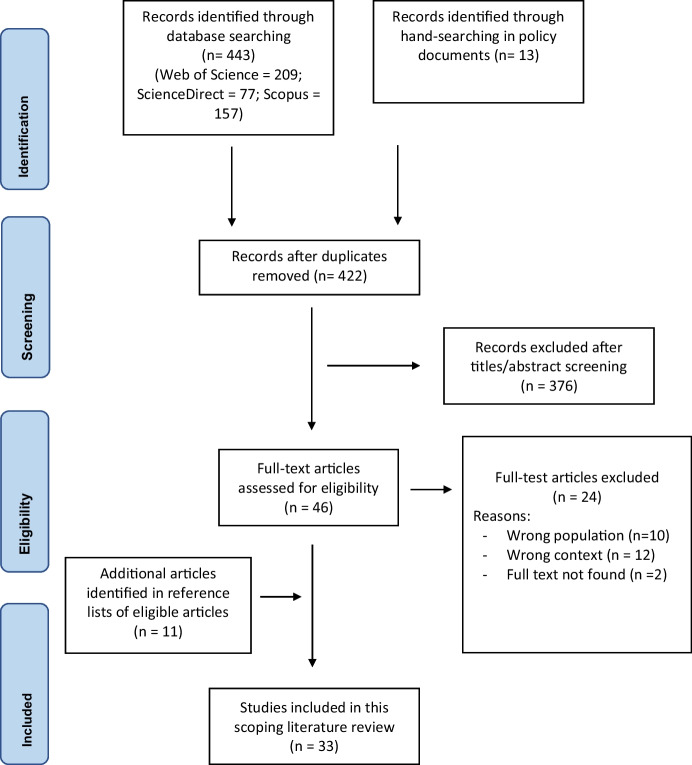
PRISMA-ScR flowchart

A total of 442 non-duplicate articles were reviewed to determine eligibility (Fig. 1). After screening, 46 sources met eligibility criteria, of which 22 were included in this review. Full-text articles were excluded because of the following reasons: full-text unavailable (*n* = 2), wrong study context (*n* = 12) (e.g., pre-COVID-19 or other geographical location), wrong population or participants (*n* = 10) (e.g., regular migrants or essential workers). Eleven eligible articles were identified by screening the reference lists of included studies. In total, 33 sources were included in this scoping review; 28 were qualitative studies, one quantitative, and four mixed methods (Table 2). The majority of the articles focused on the US and Mexico (69.7%), whereas other articles were based in Latin America and/or the Caribbean (21.3%), the Mexican-Guatemalan border (3.0%), Honduras (3.0%) and globally (3.0%). Most sources (*n* = 26) focused on irregular migrants, of which three sources specifically examined the experiences of irregular migrant families and children. The remaining sources (*n* = 7) had a broader scope and focused on general migration policies without specifically focusing on irregular migrants (*n* = 5) or did not specify the location (*n* = 2). Around half of the sources (*n* = 16) did not explicitly mention a policy name or restrictive measure, broadly referring to policy decisions as “border closures.” The remaining sources provided the policy names, such as Title 42 in the US, Migrant Protection Protocols (MPP) MPP in Mexico, and the US-Guatemala Asylum Cooperative Agreement. COVID-19-related policies or restrictions were mentioned in almost all sources (*n* = 30), and fewer studies reported migration flows (*n* = 23) and migrant experiences (*n* = 22). Overall, 14 studies reported information on all three categories.

**Table 2:** Characteristics of included articles

First author	Document type	Study location	Policies and/or restrictions	Migrant characteristics	Migrant experiences
Araujo and Sarmiento (2021)	Empirical research	Latin America	U.S. anti-immigrant policies such as Family Separation, Migrant Protection Protocols, and Title 42, U.S. court closures due to COVID-19	Central American migrants in US and Mexico, Guatemalan migrants at the Southern Mexican border	Family separation, violence, confinement, sexual assault, risk of contracting SARS- CoV-2, physical and psychological disorders
Astles (2020)	Commentary	Central America and Mexico-U.S	Mexican transit policies	Guatemalan, El Salvadoran, and Honduran migrants in Mexico	Violence, hurricanes, hunger, poverty in the home and transit countries
Blue et al. (2021)	Empirical research	Mexico	Migrant Protection Protocols, Third-country bilateral agreements such as Asylum Cooperative Agreement between the U.S. & Guatemala, Title 42, and COVID-19 lockdown measures	Central American migrants in Mexico	Prolonged waiting times for asylum, confined, isolated and unsanitary living conditions
Bojórquez et al. (2021b)	Empirical research	Mexico	Migrant Protection Protocols, Title 42, U.S. court closures	Mexican Migrants the U.S.-Mexican border	Mental disorders, fear, violence in refugee shelters, protracted displacement
Bonfiglio et al. (2020)	Commentary	Mexico and the U.S	-	-	Stress and anxiety disorders, improper treatment of pre-existing comorbidities
Rabasa Jofre (2021)	Information tool	Latin America and the Caribbean	Border closures in all Latin American countries	-	-
Clark and Patterson (2020)	Commentary	Latin America	Delayed COVID-19 lockdown measures	Central American migrants in Mexico	-
Crawley (2021)	Empirical research	Global	U.S. border closures, Title 42, rapid expulsion procedures at the southern border due to COVID-19	All asylum seekers at the US-Mexican border	-
ECLAC (2020)	GovernmentReport	Latin America and the Caribbean	Increased border control measures in Costa Rica, Guatemala, El Salvador, Migrant Protection Protocols	Central American migrants at the US-Mexican border	The conditions at points of departure in Northern Central America: violence, political instability, economic crisis, poverty and inequality During transit through Mexico: citizen insecurity, organized crime, extortion and risks to the health and lives Upon arrival in the US: vulnerability, racism and xenophobia
Ernst (2020)	Commentary	Central America and the U.S	Border closures and lockdown measures in Central America	Central American migrants	Fear, protracted displacement
Fonseca (2020)	Commentary	Honduras	Wall-building and detention centres leading to increased security along the U.S.-Mexico border, The construction of a military airbase along the Costa Rican-Nicaraguan border to deter migration	Honduran, Haitian, and Venezuelan migrants in transit through Mexico	-
Garrett (2020)	Essay	U.S.-Mexico border	Border control and wall-building along the U.S.-Mexico border, U.S. zero-tolerance policy, Migrant Protection Protocols, and U.S.-Guatemala Asylum Cooperative Agreement	Guatemalan, El Salvadorian, and Honduran migrants at the U.S.-Mexican border	Unsafe refugee shelters, flights with Honduran and Salvadoran migrants to Guatemala, exposed to violence in transit
Gonzalez Martin (2021)	Essay	U.S.-Mexico border	Border closure in Mexico and assisted voluntary returns	Central American migrants in immigration stations and in detention centres in Mexico	Violence and crime in detention centres, poor sanitary facilities and increased risk of contracting SARS- CoV-2
Hernandez-Arriaga and Domínguez (2020)	Empirical research	U.S.-Mexico border	Migrant Protection Protocols, Title 42, and US court closures	Central American migrant children and adults returned to Matamoros and Ciudad Juarez, Mexico to seek housing	Fear, nightmares, broken hearts, sorrow and unhappiness
IOM (2021)	Government report	Latin America and the Caribbean	International and national COVID-19 restrictions, including border controls and quarantine mandates	African refugees in Panama Honduran, El Salvadoran and Guatemalan migrants at the U.S.-Mexico border	-
IOM (2020)	Government report	Central America and Mexico	COVID-19 border closures in all Latin American countries, including Mexico and the U.S	Central American migrants in Latin America, Mexico and the U.S	-
Kauffer (2020)	Commentary	Mexico-Guatemala border	Border control in Mexico and Guatemala	Central American migrants in Mexico	-
Lara-Valencia (2021)	Empirical research	U.S.-Mexico border	U.S. border control and restrictions under Title 42,	All irregular migrants along the US-Mexican border	-
Love (2020)	Commentary	Mexico	-	Central American migrants in Mexico	Fear of contracting SARS- CoV-2 and sexual or physical assault, lack of food or humanitarian workers
MacLean (2020)	Empirical research	U.S. detention centres	U.S. zero-tolerance policy	Migrants from the Northern Triangle residing in U.S. detention centres	Unaccompanied children experience developmental, emotional and mental difficulties for long periods after reunification
Meissner (2020)	Policy brief	U.S.-Mexico border	Migrant Protection Protocols and Title 42	Guatemalan, El Salvadoran and Honduran migrants in the U.S. and Mexico	Tear gas and pepper spray to deter dissent, detainees have long lasting psychological harm and health risks
Meyer and Isacson (2019)	Empirical research	Northern and Southern Mexican border	Deployment of Mexican National Guard, U.S.-funded detention centres along Mexico’s Southern border	Central American migrants in Mexico	-
Rabasa Jofre (2021)	Commentary	Mexico	-	Honduran migrants in Mexico	Direct violence (gangs, crime and domestic) and structural violence (racism, xenophobia, poverty)
Ramirez-Garrcia and Ascencio (2021)	Empirical research	Mexico and U.S	U.S. border closures due to COVID-19, court closures, Title 42 and detention centres along the U.S.-Mexico border	Central American migrants in Mexico and Mexican migrants in the U.S	No access to medical care, limited resources and poor living conditions in Mexican detention centres
Reynolds et al. (2021)	Empirical research	Mexico	Migrant Protection Protocols, border closures, and lockdown measures	Central American migrants in Mexico	Poor mental health, distrust between migrants and aid workers, fear of contracting SARS- CoV-2
Rodríguez-García-de-Cortázar et al. (2021)	Essay	Global	COVID-19 lockdown measures and border closures	Global racial and ethnic minority migrants	Physical and linguistic barriers for migrants to obtain asylum information, and protection against SARS- CoV-2
Salas et al. (2020)	Government report	U.S.-Mexico	Migrant Protection Protocols, Title 42, Mexico’s COVID-19 policies	Mexican and Central American migrants in Northeast Mexico	Overcrowded living conditions in detention centres resulted in migrant protests
Selee (2020)	Empirical research	U.S.-Mexico	Increased border patrol in Southern Mexico, Migrant Protection Protocols, Third country bilateral agreements	Guatemalan, El Salvadorian, and Honduran migrants in Mexico	-
Shadid and Sidhu (2021)	Empirical research	U.S. detention centres	The release of all detained children from family centres and U.S. zero-tolerance policy	Central American migrants in U.S. detention centres	Mothers and children experience high rates of stress, despair, worry and panic. Children displayed peer social problems, developmental problems and poor mental health
Soto (2020)	Policy brief	U.S.-Mexico	Migrant Protection Protocols, Transit-Country Asylum Ban, Asylum Cooperative Agreement, border closure, Title 42, increased border security in Southern Mexico	Irregular migrants in Mexico	Migrants in Matamoros and Tamaulipas are threatened by physical violence, with reports of murder, torture, rape, and kidnapping by MPP migrants
Torre-Cantalapiedra (2021)	Empirical research	U.S.-Mexico	Migrant Protection Protocols, COVID-19 border closures	Guatemalan, El Salvadorian, and Honduran migrants in Mexico and the U.S	Irregular migration due to Hurricane Eta and Lota
Vera Espinoza et al. (2020)	Commentary	Latin America	COVID-19 border closures, lockdown and social distancing measures	Central and South Americans in Mexico	Overcrowded living conditions, limited access to health services and legal protection
Villamar (2021)	Empirical research	Latin America	Border closures and increased border security at Mexico-Guatemala Border and the U.S.- Mexico Border	Central American migrants at the southern Mexican border, Nicaraguan migrants in Costa Rica	Violent protests and experiences of distress in Mexican detention centres

## Results

Three thematic findings were identified in this review: 1) border closures to halt migration flows in Central America and Mexico, 2) delays in asylum procedures and subsequent consequences, and 3) migrant wellbeing in detention centers. Together, these themes illuminate how COVID-19-related policies impacted irregular migration flows and its affect on migrants with limited mobility.

### Border Closures to Halt Migration Flows in Central America and Mexico

Several articles detailed swift border closures to halt irregular migration into Mexico and eventually deter migration into the US. In the summer of 2019, before the COVID-19 pandemic, Mexico dispatched 10,000 troops of their National Guard to the southern border to police migrant entry. For comparison, there were 15,000 troops at the US-Mexican border, showing a clear resemblance to the guarded northern border with the US (Selee, 2020). Funded by the US, Mexico constructed communication towers along their southern border and installed biometric equipment in all migrant detention centers to increase Mexico’s capacity to process and receive asylum seekers (Meyer & Isacson, 2019).

In March 2020, 10 months after the signing of the US-Mexico Joint Declaration, the COVID-19 pandemic became the basis for restricting non-essential travel into the US (Soto, 2020). The US blocked the entry of all irregular migrants, asserting that asylum seekers were a threat to public health (Blue et al., 2021; Crawley, 2021). The travel restrictions and health and sanitation measures implemented by national governments limited irregular migration flows between Mexico and the US (MDP, 2021). The US used the threat to public health to enact Title 42, which directs border patrol agents to refuse entry of asylum seekers into the US (Blue et al., 2021). Simultaneously, the US considered irregular migrants on the border as non-essential personnel, thereby authorizing forceful deportations into Mexico (Villamar, 2021). To illustrate, the US deported an estimated 40,000 irregular migrants during the first 4 months of the pandemic (Ramirez-Garrcia & Ascencio, 2021).

Many Latin American countries enacted travel and movement restrictions for citizens and international travelers in response to the COVID-19 pandemic (IOM, 2021). In the Americas, 92% of countries in the region enacted public health-related travel restrictions and closed their borders, leaving irregular migrants stranded in transit countries in the Northern Triangle and predominantly in Mexico (IOM, 2020). Mexican authorities reported 56,622 irregular migrants between January and April 2021, of whom 94% originated from the Northern Triangle (MDP, 2021). In Central America, several irregular immigrants were left legally unprotected or forced to return to the dangers of their home country because of COVID-19 policies (Bojórquez et al., 2021b). For example, the Costa Rican government created a military airbase as a public health measure to deter the entrance of non-citizens (Fonseca, 2020). This military station was located at the border with Nicaragua and prevented the influx of irregular migrants, as Nicaraguans constitute 10% of Costa Rica’s current population (Fonseca, 2020; Clark & Patterson, 2020). In Honduras, public health-related travel restrictions and subsequent border closures led to the postponement of caravan journeys, so individuals were continuously exposed to direct violence from gangs and crime organizations (Rabasa Jofre, 2021; IOM, 2021). El Salvador also closed its airports to passenger flights and its land borders as a public health protocol (ECLAC, 2020).

The Guatemalan government also enacted public health-related travel restrictions and deployed their military to block all land entry for non-citizens, which subsequently increased the number of informal crossings by circumventing immigration protocols and officials (Kauffer, 2020). Moreover, on March 17, 2020, Guatemala announced that it would suspend receiving deported Hondurans from the US as part of their public health measures (Ernst, 2020). Guatemalan authorities denied entry of Instituto Nacional de Migracion (INM) buses, forcing Mexican authorities to release 480 Central American migrants in the southern state of Chiapas, Mexico. These migrants faced harassment and were attacked by local citizens over the fear of contracting SARS- CoV-2 (Soto, 2020). The accumulation of migrants from Central America and the presence of COVID-19 has increased xenophobia among Mexicans, resulting in many migrants facing discrimination and altering their accents to mimic local Mexican accents (Torre-Cantalapiedra, 2021; Barajas et al., 2021). Araujo and Sarmiento (2021) described that the entry of irregular migrants and mostly Guatemalan immigrants were violently blocked by armed forces at the Southern Mexican border. In addition, Villamar (2021) describes thousands of Central American migrants deported from southern Mexico, but many were unable to return home due to border closures (Villamar, 2021). Similarly, 90% of the asylum seekers at the US-Mexican border were left legally unprotected and immobilized due to travel restrictions (Reynolds et al., 2021). By mid-2021, only a handful of border closures remained worldwide, and almost all internal restrictions were lifted (IOM, 2021).

### Delays in Asylum Procedures and Subsequent Consequences

Starting in April 2019, Mexico implemented a restrictive migration policy to prevent the transit of migrants through the country (Astles, 2020). Although the number of irregular migration policies in Mexico increased, the government has been slow to implement policies to protect the health of asylum seekers in-transit migrants (Bojórquez et al., 2021b). Moreover, the onset of the COVID-19 pandemic triggered delays and terminations of asylum procedures. In Mexico, the Comisión Mexicana de Ayuda a Refugiados (Mexican Commission for Refugee Assistance) suspended their 45-day processing due to lockdown measures (Soto, 2020). Similarly, because of lockdown measures, asylum procedures were delayed or suspended in the US (Vera Espinoza et al., 2020). Court closures under Title 42 meant that asylum seekers that arrived at the US-Mexico border were not placed under the Migrant Protection Protocols (MPP); rather, they were expelled into Mexico without due processing (Blue et al., 2021; Hernandez-Arriaga & Domínguez, 2020). This resulted in makeshift camps of thousands of migrants along the plaza that is adjacent to the international bridge in the border city of Matamoros, Mexico (Blue et al., 2021).

The implementation of the MPP alongside court closures due to the COVID-19 pandemic left thousands of migrants stranded in Northern Mexico (Salas et al., 2020). The situation was further exacerbated by shelters closing or reducing their capacity to mitigate the spread of SARS-CoV-2 and the heightened influx of irregular migrants (Vera Espinoza et al., 2020). These actions left many migrants homeless and living in precarious conditions (Vera Espinoza et al., 2020). Additionally, local soup kitchens were forced to close due to COVID-19 restrictions. In Sonora, Mexico one soup kitchen altered their service delivery and addressed migrant needs by creating to-go packages to satisfy social distancing regulations (Lara-Valencia and García-Pérez, 2021). Following court closures and shortages of shelters, many asylum seekers have attempted swimming across the Rio Grande, but few have been successful and numerous deaths were reported (Hernandez-Arriaga and Domínguez, 2020). Furthermore, migrants trapped in-transit destinations were exposed to violence like kidnapping, rape, and robbery (Garrett, 2020). Likewise, according to the IOM (2020), human smuggling persisted during the pandemic; however, it is unclear to what extent human smuggling persisted and whether it differed from pre-pandemic times.

Furthermore, the US accelerated its deportation process of irregular migrants into Mexico during the COVID-19 pandemic, directly increasing migrants’ vulnerability to SARS-CoV-2 (Rodríguez-García-de-Cortázar et al., 2021). The US has been accused of deporting migrants infected with SARS-CoV-2 without providing essential medical care. This is due to the absence of COVID-19 protocols during deportation and the additional health risks individuals experience while migrating irregularly (Ramirez-Garrcia and Ascencio, 2021). In relation to deportation, irregular migrants have continued to face racism, xenophobia, and now discrimination over the perception of being carriers and bringing the SARS-CoV-2 virus into Mexico and Central America (ECLAC, 2020). Migrant caravans that emerged in Central America in 2021 faced criminalization because they were perceived as a threat to public health (González Morales, 2021). In one instance, a migrant caravan from Honduras was violently expelled by the Guatemalan army resulting in injuries (González Morales, 2021). The COVID-19 pandemic increased the complexity of seeking asylum due to the added criminalization for irregularly crossing borders that were closed due to public safety. Therefore, in 2020, there was a steep decline in irregular migrant caravans coming from Central America due to the halt of asylum processing and COVID-19 related border restrictions (Ernst, 2020).

### Migrant Wellbeing in Detention Centers

Irregular migrants attempting to cross the US-Mexico border were captured and placed in one of the 137 detention centers (DCs) present in these two countries (Araujo & Sarmiento, 2021). DCs are informally referred to as “hieleras” (ice boxes), “metal cages” or “perreras” (dog kennels), reflecting their incarcerated character (Bonfiglio et al., 2020; Garrett, 2020). In Mexico, 65 DCs exist where the majority of the detainees originate from the Northern Triangle (Gonzalez Martin, 2021). Between January 2019 and 2020, the number of detainees increased by 5,000 in Mexico (IOM, 2021). With Mexico’s sharp increase in migrant apprehensions, many of the detention centers are operating beyond capacity, with an average of 61% more migrants and, in some cases, over 300% capacity (Meyer and Isacson, 2019).

Due to isolation, solitary confinement, violence, and overcrowded living conditions in DCs, pre-existing mental disorders worsened during the COVID-19 pandemic (Araujo & Sarmiento, 2021). Detainees that experienced kidnapping or detention before arriving at the DCs felt specifically anxious and unsafe in the living conditions that arose during the lockdown (Bojórquez et al., 2021a). Some centers provided online dance classes to mitigate adverse mental health effects; however, the effects of these classes were not reported (Bojórquez et al., 2021a). Furthermore, several studies reported limited resources, like the inability to access healthcare, lack of personal protection equipment, and confined and crowded living spaces in DCs during the lockdown (Hernandez-Arriaga and Domínguez, 2020; Ramirez-Garrcia and Ascencio, 2021; Villamar, 2021). These conditions caused feelings of fear of contracting SARS-CoV-2, resulting in anxiety and anger amongst migrants within the DCs (Love, 2020; Rodríguez-García-de-Cortázar et al., 2021; Villamar, 2021). In March 2020, migrants in three National Institute of Migration (NIM) in Mexican detention centers organized a hunger strike and protested the overcrowding and poor sanitary conditions that appeared to be exacerbating the spread of the SARS-CoV-2 virus (Soto, 2020). As a response to protests, US and Mexican officials responded violently, using tear gas and pepper sprays to minimize and discourage dissent (Meissner, 2020; Soto, 2020).

In 2020, the US mandated the release of irregular migrant children from residential centers; however, their release was unaccompanied as their parents remained in US custody (Shadid and Sidhu, 2021). The intention of the forced family separation under the US’ Zero Tolerance Policy was to discourage migrant families from the Northern Triangle from entering the US (Garrett, 2020). The practice of family separation continues to be a long-standing immigration tactic employed by the US to discourage migration, which started as early as the 1920s (Schacher, 2020). A cross-sectional study by MacLean et al. (2020) examined the effects of forced family separation on children at the US-Mexican border and found an increased vulnerability of separated migrant children from the Northern Triangle according to their mothers. These children showed peer problems (21%), general difficulties (15%), and almost half experienced “abnormal” emotional problems (MacLean et al., 2020). Moreover, children from a similar sample demonstrated attachment difficulties, developmental relapses, and the inability to sleep alone (Shadid & Sidhu, 2021) . In addition, a study by Hernandez-Arriaga and Domínguez (2020) depicts testimonies of migrant children in migrant camp Matamoros. Children made drawings of their experiences in the camp, all portraying unhappiness, worry, nightmares, and isolation. Likewise, the majority drew “la migra” (the US-Border Patrol) and how the immigrant authorities made them feel unwanted (Hernandez-Arriaga and Domínguez, 2020). Another study on Matamoros describes that migrant women were raped and that individuals from the LGTBQ + community were beaten mercilessly in the DCs during the pandemic (Blue et al., 2021).

## Discussion

Prior to the COVID-19 pandemic, the US implemented numerous policies to deter irregular migration, such as “Metering” (Leutert, 2020a, 2020b), the Migrant Protection Protocols (MPP) agreement with Mexico (Soto, 2020), and the “Third-Country Asylum Rule” denying entry to asylum seekers who transited through Mexico or other Central American states without claiming asylum and receiving a negative decision (DHS, 2019). In 2018, the migrant caravans from Central America and the economic pressure from the US resulted in Mexico restricting irregular migration through their southern border (Vega, 2021). Together, these policies illustrated growing efforts to exclude asylum seekers from crossing the US border prior to the COVID-19 pandemic (Gilman, 2020). During the pandemic, US border closures and the implementation of Article 42 were followed by the continuation of forceful deportations that triggered similar migration policies and restrictions in Mexico and Central America (Ramirez-Garrcia and Ascencio, 2021). Though border closures were enacted as a COVID-19 public health response, this scoping review supports scholars’ argument that these policies were primarily implemented to prevent irregular migration flows (Sanchez, 2020).

ThXis review set out to examine how COVID-19-related policies and restrictions in Central America, Mexico, and the US impacted irregular migration flows to the US-Mexico border, and to document the experiences of irregular migrants stranded in Central America and Mexico as a result of these interregional COVID-19 policies and restrictions. This scoping review demonstrates that migration policies used to justify border closures and halt migration flows in Central America and Mexico were an extension of pre-pandemic efforts to limit migration. As our review highlighted, the border closures in Costa Rica, El Salvador, Guatemala and Honduras, the restriction of movement within Mexico and along the US-Mexico border illustrated how public health measures were used to justify the travel restrictions to minimize the spread of SARS-CoV-2.

Further, the findings highlight which obligations in the Migrant Protection Protocols (MPP) were prioritized. Mexico strengthened migration control at the Mexico-Guatemala border and accepted more non-Mexican asylum seekers deported by the US, as outlined in the protocols; however, humanitarian protections were not increased and the US did not expedite processing asylum cases. Rather, opposite outcomes were identified during the pandemic, highlighting how the MPP obligations have been selectively prioritized to deter migration and satisfy the US’s migration policy interests. The fulfillment of the remaining obligations could not be identified within the scope of this review; however, it is important to note that the damage done to the asylum process by the Trump administration in the US has not been restored by the Biden administration (Kocher, 2021). Despite the top scientists from the Center for Disease Control (CDC) calling for an end to Title 42 and President Biden declaring the pandemic “over”, the current administration continues to deny entry to asylum seekers using Title 42 (Beitsch & Bernal, 2022). Furthermore, the Biden administration plans to expand the US’s restrictive immigration policies at its Southern Border and extend the dial of entry Cuban, Haitian, and Nicaraguans entering the US through Mexico (AP, 2023).

Following in the trend of increasingly restrictive migration policies within Central America, Mexico and the US, the delays in processing asylum cases prior to the pandemic (e.g., metering and the Third-Country Asylum Rule) also increased during the border closures in 2020, resulting in further delays and suspension of asylum cases. Rather than migrants being placed under MPP, an initial response during the pre-pandemic period, the US accelerated deportations and expulsions from the US-Mexico border and many asylum seekers were stranded in transit destinations where they were exposed to violence, kidnapping, robbery, and sexual abuse (Garrett, 2020). The policy actions that precipitated asylum processing delays followed a similar trend of border closures, whereby asylum processing was significantly reduced during the Trump Administration and the pandemic became a justification to effectively halt processing and increase deportation of vulnerable individuals and families.

The restrictive migration policies and delayed asylum processing had significant consequences for the health and wellbeing of migrants. Several individuals that remained near the US-Mexican border and Mexico were placed in unsanitary and overcrowded detention centers, which exacerbated their mental and physical wellbeing, increased fear of contracting the virus and heightened their exposure to violence (Bonfiglio et al., 2020; Gonzalez Martin, 2021). Migratory routes through Northern Africa to Europe have reported similar trends in protracted displacement, whereby border closures have led to dangerous deportations, stranded migrants, violent attacks by local communities and individuals living in inhospitable conditions (United Nations, 2021). Our review reveals similar experiences and also highlights how residing in detention centers may harm a migrant’s already fragile mental and physical wellbeing. A systematic review by Filges et al. (2018) also acknowledges the increased risk of mental health disorders like post-traumatic stress disorder (PTSD), depression, and anxiety amongst several migrants in detention centers across the globe. Border closures, delays in asylum processing, deportation and expulsions may have a cumulative negative effect on migrant mental and physical wellbeing. This impact is especially important to note for children’s development, as research has reported emotional and physical problems among unaccompanied migrant children in US detention centers (Linton et al., 2017).

Literature on the experiences of irregular migrants in this region remains limited. Most of the included literature that explored migrant experiences was in relation to the economic conditions of irregular migrants in the region. Since irregular migrants may be unable to access legal status or employment or any form of social service, irregular migrants succumb to harmful living conditions like the ones described in this review (Echeverría, 2020). Notably, within this review, migrant experiences are often impersonal, generalized, and communicated by policymakers, health professionals or volunteers instead of first-person accounts from irregular migrants (Love, 2020; Vera Espinoza et al., 2020) .

### Strengths and Limitations

This is the first scoping review to investigate the impact of COVID-19-related policies on irregular migration in Central America. One of the strengths is that the review uses the JBI manual for designing and conducting a systematic methodology. Secondly, data were extracted and analyzed independently by two graduate-level reviewers, one Dutch and one Mexican–American, with clear rules to consult doctoral-level English and Spanish-speaking contributors in case of disagreement. The reviewers and contributors identified this review as a pressing topic in migration and public health policy debates, inspired by personal experience of migration and scholastic interests. Third, the sources included in this review were in English and Spanish, allowing the authors to identify and include relevant documents from Central America and Mexico. Lastly, an exhaustive systematic search strategy was adopted to identify relevant sources in several literature and policy databases.

Despite these strengths, this scoping review has some limitations. Given the vast scope of possible literature sources, it is probable that not all relevant sources were included in this review. As COVID-19 and its impact on irregular migration is an ongoing phenomenon, there may be recently published documents not captured in this review. Few studies distinguished between groups with different migration backgrounds, and thematic analysis could not identify the experiences among different irregular migrant groups. Lastly, several documents in this review did not include the exact names of the migration and COVID-19 policies and referred to restrictive measures broadly, such as “policies” or “lockdown measures.” The lack of specificity in naming policy measures hindered the study authors’ ability to identify trends on the direct impact of policies on the experiences of irregular migrants. Finally, several of the included studies conceptualized irregular migrants as a homogenous group and authors were unable to examine migrant experiences by migrants’ country of origin.

### Implications for Future Policy and Research Efforts

The COVID-19 pandemic disproportionately affects the lives of thousands of irregular migrants in Mexico and Central America. Moreover, the reported experiences of irregular migrants outline several important implications. First, several countries in a somewhat coordinated effort attempted to halt migration through various policies under the guise of preventing the spread of SARS-CoV-2. Upon review, such policies failed to prevent the spread of SARS-CoV-2 and further harmed irregular migrants through border closures, halts to asylum procedures, and failure to improve living conditions in shelters and detention centers. Instead, these policies reaffirmed and created new barriers for irregular migrants. Future migration policies in the Americas require multilateral agreements that prioritize the Universal Declaration of Human rights. These policies should ensure protection for individuals seeking asylum, and public health measures in accommodations for irregular migrants. Local government agencies and non-governmental organizations should work cooperatively to provide safe housing and accommodations for asylum seekers in transit. Further, multilateral discussions are needed on how to address the social and environmental conditions that force individuals to leave their country of origin, which may contribute to decreasing future irregular migration flows throughout the region. Though multilateral refugee agreements such as the MPP consider some of the recommendations presented here, measurable outcomes such as refugee wellbeing, quality of accommodations, ability to gain employment and additional indicators of refugee resettlement should be the benchmark for successful implementation of such policies.

Sustaining international collaboration among Central American states, Mexico, and the US requires a critical policy analysis that takes into account the numerous national and international actors and their political and economic interests in addressing irregular migration within the region. Looking to other international multilateral refugee agreements, such as the EU-Lebanon Compact (Pelayo, 2018) and the UK-Rwanda Asylum Plan (formally called the Migration and Economic Development Partnership with Rwanda; Gower & Butchard, 2022), we see that the country with the highest economic and political power creates the conditions set out in these agreements. Further, the rising xenophobia and its role in shaping popular political discourses has also influenced political decisions regarding refugee resettlement programs (Bose, 2022).

However, the Nansen Initiative presents a possible solution to improving refugee protection and resettlement. The Nansen Initiative is a “state-owned” process, requiring action in three areas: 1) enhance methodological approaches, data collection and analysis of disaggregated data to better capture the demographics of asylum seekers and intentions to continue transiting; 2) improving humanitarian protection measures and creatively utilizing existing legal frameworks for individuals in need; and 3) addressing migration-related risks within countries of origin to allow for planned and legal migration, allowing relocation to occur in a safe manner (Martin, 2016). Though this approach was presented within a natural disaster and climate change context, Martin (2016) suggestion can equally be applied to the context of forced displacement in Central America given that violence and political instability are also crises. Anticipated improvements in migration flows and subsequent migrant experiences are therefore predicated on the cooperation of all Central American states, Mexico and the US and future multilateral agreements should seek to incorporate the three principal areas set forth in the Nansen Initiative, or similar approaches that consider the human experience in refugee resettlement initiatives.

This review also highlighted how these policies also exacerbated health inequities for irregular migrants, revealing that migration policies have not considered nor taken responsibility for the health needs of irregular migrants. Policies implemented to manage irregular migration flows should consider the health implications of existing infrastructures and implement health and safety measures that address current public health concerns and migrant wellbeing. This review reveals that the protracted displacement experienced by irregular migrants throughout Central America and Mexico may increase their risk of developing mental health issues. Refugees and asylum seekers experience post-traumatic stress disorder (PTSD), anxiety, and depression over time, and many continue to experience mental health issues for years to come (Henkelmann et al., 2020). These issues may further be exacerbated by the lack of access to healthcare services and psychosocial support. The mental health needs of asylum seekers should be prioritized in future research and practice, for example, where refugee community-based organizations can utilize low-cost interventions like psychological first aid in the delivery of mental health support. Additional recommendations include the provision of free public healthcare services to asylum seekers, mitigating structural barriers that hinder access to care, and increasing awareness of existing health services offered through local non-governmental organizations.

Future research should consider the diversity of migrants groups and how these vary during transit and at their final destination. Future research should also examine the relationships between discrimination and subjective wellbeing along migratory routes in Central America. By capturing experiences along common routes, policymakers, practitioners, and humanitarian organizations can address and mitigate the risks of irregular migration, including precarity and instances of violence.

## Conclusion

Multilateral refugee agreements in Central America, Mexico, and the US have prioritized national interests and have overlooked the public health concerns of the asylum seekers at their borders. The restrictive migration policies implemented before and during the COVID-19 pandemic along with the increased militarization of several Central American borders reveal how asylum seekers have not been sufficiently considered in the creation and implementation of public health-related policies. The conditions in which asylum seekers are fleeing are public health issues, whereby exposure to violence and humanitarian disasters in countries of origin remain a priority area of concern, which has only been exacerbated by the COVID-19 pandemic. This review emphasizes the importance of distinguishing immigration policy from public health policy and the need to prioritize appropriate and effective implementation of these policies. The interplay between public health priorities and forced displacement require a coordinated response that take into account social, public health and migration policies. By doing so, a multilateral public health and refugee resettlement agreement has the potential to improve the conditions within countries of origin and the capacity to adequately provide resources for asylum seekers in countries of destination. The health inequities exacerbated by these policies will not be easily resolved with changes to immigration policies alone, however, but will require a multifaceted approach to ameliorating health for such a vulnerable population. Future multilateral agreements should clearly outline social and environmental indicators as a benchmark for successful implementation, and create risks assessments and strategies to anticipate unprecedented shifts in migration flows. By examining how national political interests and popular political discourses shape multilateral refugee agreements amidst public health crises, we gain a better understanding of the context surrounding interregional and extra-continental migration flows and its impact on overall migrant wellbeing.
